# Effect of oral administration of GABA on thermoregulation in athletes during exercise in cold environments: A preliminary study

**DOI:** 10.3389/fnut.2022.883571

**Published:** 2022-07-15

**Authors:** Hongli Wang, Lin Cheng, Yanbai Han

**Affiliations:** College of Physical Education and Health, Guangxi Normal University, Guilin, China

**Keywords:** γ-aminobutyric acid, nutritional supplements, thermoregulation, exercise, cold environment

## Abstract

**Background:**

γ-aminobutyric acid (GABA), a common ingredient in sports supplements and other health products, regulates body temperature in the preoptic area and anterior hypothalamus (PO/AH). To date, no study has examined the effect of GABA on thermoregulation during exercise in humans in a cold temperature environment (11 ± 0.3°C, 45% ± 2% relative humidity).

**Methods:**

We performed a randomized, double-blind study. Ten trained male athletes consumed either a drink (3 ml/kg weight) containing GABA (1,000 mg, trial G) or an equivalent amount of placebo drink (trial C) before exercise. They rested for 20 min and then cycled at 60% of maximum output power for 40 min, pedaling at 60 rpm, and recovered for 20 min. Core temperature (T_c_), skin temperature (upper arm, chest, thigh, calf), and heart rate (HR) were monitored at rest (T_0_), exercise begins (T_20_), 20 min of exercise (T_40_), the exercise ends (T_60_), and at recovery (T_80_).

**Results:**

Compared to T_0_, T_c_ decreased significantly at T_20_ and increased significantly at T_40_, T_60_ and T_80_ (*p* < 0.01). From 35–80 min, the T_c_ was higher in trial G (peaked at 37.96 ± 0.25°C) than in trial C (37.89 ± 0.37°C), but it failed to reach significant difference (*p* > 0.05); T_sk_ continued to increase during exercise and was significantly higher than T_0_ at T_40_ (*p* < 0.05), T_60_ and T_80_ (*p* < 0.01). There was no significant difference in T_sk_ between the two trials (*p* > 0.05).

**Conclusion:**

Our findings provide initial evidence that oral administration of GABA does not affect thermoregulation and has no adverse effects on the body as an ergogenic exercise supplement during exercise in cold environments.

## Introduction

Major sporting events such as the Winter Olympic Games and Winter Paralympic Games are held during the cold winter season, with athletes inevitably training and competing in cold conditions. For many athletes, participation in sport (e.g., skating, skiing, and running) in cold environments is part of daily life. Studies have shown that ambient temperatures of 10–16°C can induce physiological reactions such as peripheral vascular contraction and peripheral blood flow reduction to maintain the body heat balance (1). In a cold environment, the body's energy expenditure increases, resulting in excitation of the sympathetic nervous system and higher catecholamine concentrations. These changes cause vasoconstriction of the terminal blood and skin vessels, increased heart rate (HR) and cardiac output, decreased skin and limb sensitivity, reduced skeletal muscle contractility and coordination, and reduced joint mobility. Prolonged exercise (1–4 h) in cold weather may result in excessive heat loss from the body, affecting thermoregulation and resulting in hypothermia. Individuals may be at risk for thermoregulatory fatigue or even loss of life (1–4).

Human body temperature is comprised of core and shell temperatures. Core temperature (T_c_) is the temperature of the body's internal organs (e.g., heart, lungs, brain, and abdominal viscera), usually measured during exercise by rectal, esophageal, and gastrointestinal temperatures. Shell temperature is defined as the temperature of the body's peripheral tissues (skin, subcutaneous tissues, and muscles). Skin temperature, in particular, is influenced by the thickness of the skin and subcutaneous adipose tissue. It is also affected by the skin blood flow and ambient temperature and humidity (5). The preoptic area and anterior hypothalamus (PO/AH) is the thermoregulatory center of the body. Its role is to maintain a normal and relatively stable body temperature, ensuring a dynamic balance between heat production and loss (6).

γ-aminobutyric acid (GABA), with the molecular formula C_4_H_9_NO_2_, is a predominant inhibitory neurotransmitter in the hypothalamus. It was first identified in the brain in 1950, with GABAergic synapses noted as more abundant in the PO/AH than other brain regions (7–9). GABA is also widely found in plants, animals, and microorganisms. In addition to natural sources such as germinated brown rice, soybeans, fruit, and lactic acid bacteria, GABA can also be produced by chemical synthesis, plant enrichment, and microbial fermentation, allowing for better application of GABA in plants and animals. There has been widespread interest in the functional and health effects of GABA because it is a small molecular weight non-protein amino acid proved safe to eat and used in many foods and beverages (including novelty foods) in recent years. Studies have shown that oral administration of GABA reduces neuronal activity, prevents overheating of nerve cells, and has physiological effects such as preventing arteriosclerosis, reducing stress, improving sleep, enhancing exercise-induced muscle hypertrophy, regulating cardiac arrhythmias, lowering blood pressure and blood lipids and enhancing liver function. Moreover, GABA has positive effects in the treatment of psychiatric disorders, epilepsy, convulsions, Huntington's and Parkinson's disease (10–15).

Evidence from animal studies suggests a positive relationship between GABA and thermoregulation, and an understanding of the underlying mechanism is gradually emerging. Thermoregulation of freely-moving rats during pharmacological stimulation of GABA in normal (23°C), cold (5°C), and hot (35°C) environments indicated that GABA has a crucial thermoregulatory role in the PO/AH. Body temperature decreased in hot ambient environments through the central administration of GABA in animals, while it rose in cold environments (16). In humans, GABA's role in decreasing core body temperature at rest and during exercise in hot environments is well established (17). However, no studies have examined the use of GABA as a food supplement in a functional beverage that increases core body temperature during exercise in cold environments, provides effective measures to counteract cold conditions, prevents cold injury, and improves athletic performance during training and competition. Cycling in cold environments (15°C) poses a serious challenge to the human conditioning system compared with temperate environments (18–25°C), and longer exercise duration may result in a greater risk of injury (18–20). When athletes cannot increase heat production to compensate for environment-related heat loss, their endurance and performance will decrease, and the risk of injury will increase. This study investigated whether oral administration of GABA increases core body temperature in athletes in a cold environment. To clarify the effects and underlying mechanisms of GABA, we hypothesized that the systemic administration of GABA to trained athletes during exercise would induce higher core body temperature in a cold environment. To test this, we compared thermoregulatory responses in athletes after oral administration of a GABA-containing sports drink or a placebo during exercise in a cold environment over two trials. If oral administration of GABA leads to elevated human body temperature during exercise in cold environments, evidence-based practical strategies could then be used to resist the negative effects of cold environments. This would have important practical applications for ensuring safe training and improved performance in sports carried out in the cold.

## Methods

### Participants

Ten healthy male college athletes (mean ± standard deviation, age: 21.4 ± 1.0 years, height: 181.4 ± 5.2 cm, weight: 72.5 ± 5.4 kg, VO_2max_: 63.0 ± 5.7 ml/kg^−1^min^−1^) volunteered to participate in the study. The participants were middle-long distance runners who had taken part in regular sports training for a minimum of 3 years, training at least 6 h per week. The sample size calculation (effect size = 0.25, alpha level = 0.05, power = 0.80) using G^*^Power (version 3.1.9.7; Dusseldorf, Germany) indicated that 10 participants were required for this study (21, 22). The investigators enrolled participants after assessing them and based on the following criteria: (1) had no chronic diseases and cold acclimatization; (2) physical activity for at least 30 min per day, three times per week; (3) no cardiovascular, metabolic, or respiratory disease history; (4) no fever; and (5) no use of any supplements or medications that affect thermoregulation. One week before the study, participants were informed about the procedures and dangers involved and signed informed consent forms. All research protocols followed the principles of the latest declaration of Helsinki and received approval from the ethical committee of Guangxi Normal University.

### Research design

The study was conducted using a randomized, double-blind, cross-over design. Each participant underwent a familiarization session and two experimental trials in a randomized order. Participants completed two trials during the randomized second and third visits: a controlled trial (trial C) and a GABA trial (trial G). To ensure drug washout, each condition was separated by at least 4 days (23). Both trials were performed at the same time of day to minimize the influence of circadian rhythms on body temperature and other biological variables. Participants were instructed to follow the same diet on testing days to minimize the effects of nutrition. All participants were asked to abstain from alcohol, caffeine, tea (>48 h), strenuous exercise (>24 h), and eating a standard meal (>2 h) before beginning each experiment. Both trials were conducted in winter (December-January) with a room temperature of 11°C and temperature and humidity controlled by air conditioning and humidification equipment set at 11 ± 0.3°C and 45 ± 2% relative humidity.

#### Preliminary testing and familiarity

All participants completed body composition (BOD POD Body Composition Tracking System, USA) and height (SYHW-80D stadiometer, China) measurements on arrival at the laboratory for the first time. Before assessing VO_2max_ in the preliminary experiment, participants performed a 5-min warm-up. Maximum oxygen consumption was measured using a graded exercise test on a friction braked bicycle ergometer (Monark839E, Sweden). The test began at 0 kp and then increased load by 0.25 kp every 15 s while maintaining a pedal frequency of 60 rpm until volitional exhaustion. Exhaustion was characterized as the condition when participants rated perceived exertion at 19-20 on the Borg scale, pedal frequency was <60 rpm for more than 5 s despite verbal encouragement, and HR reached a maximum value (220-age). A week before the experiments, participants were familiarized with the equipment and procedures involved in the study. The seat heights, bar heights, and positions on the cycle simulators were adjusted independently for each participant prior to the test, and the settings were recorded so they could be reproduced in each subsequent experimental trial.

#### Drug treatment

The participants were given GABA (1000 mg) dissolved in 3 ml/kg body weight of sports drink (GABA drink) in trial G or an equivalent amount of sports drink (placebo) in trial C. The GABA (500 mg/tablet, Swanson) doses followed previous research (23–25). Participants were randomly assigned to receive either a placebo or GABA drink on the first visit. The other drink was given at a second visit. The temperature of both placebo and GABA drinks was 8°C at the time of drinking. The sports drinks (MI zone beverage, Danone, China) contained 88 KJ of energy, 4.8 g of carbohydrates, and 0 g of fat and protein.

#### Exercise protocol

The participants performed at 60% of the maximum load for 40 min at a speed of 60 rpm (23). The mean load of the participants was 2.0 ± 0.3 kp.

#### Procedures

**Figure 1:**
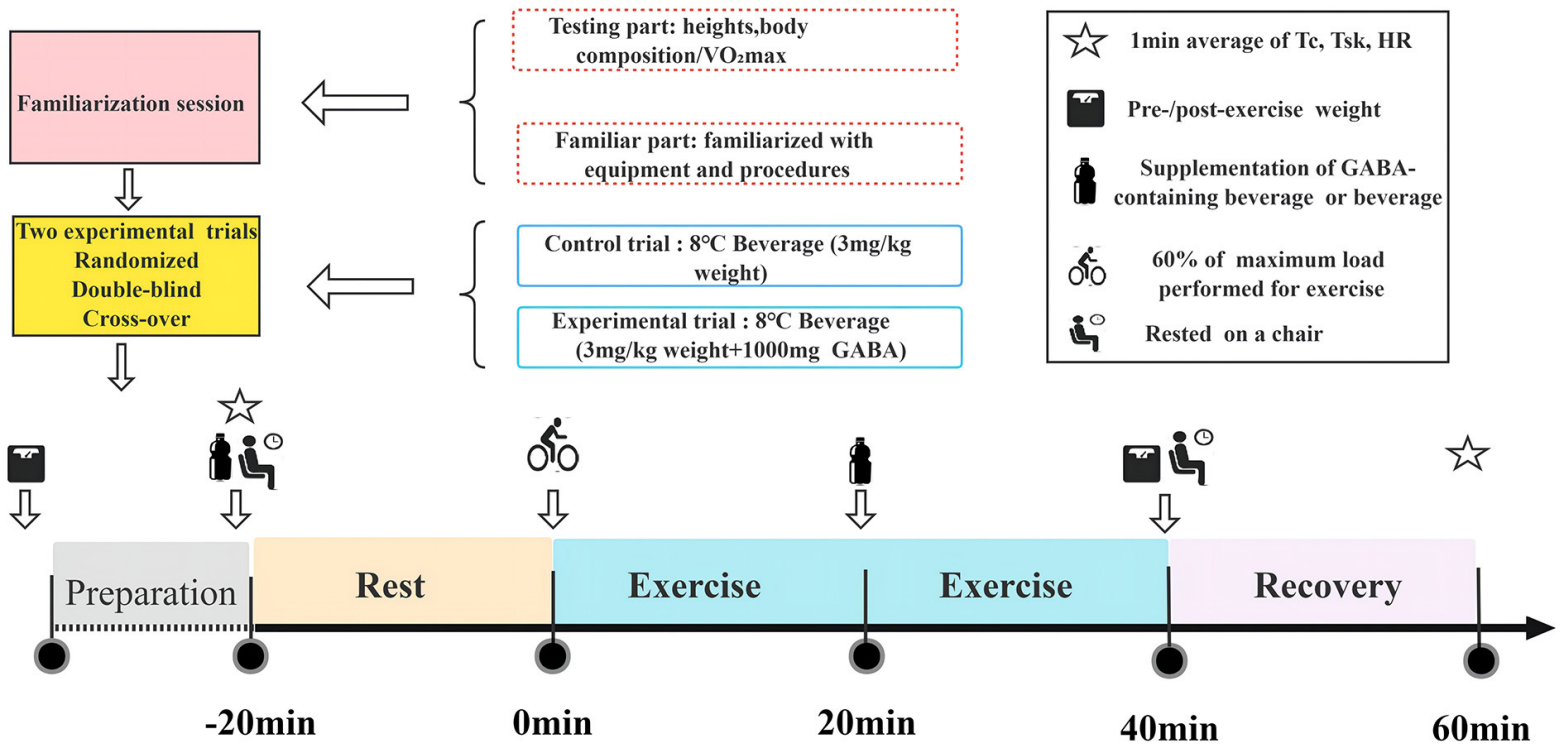
Flow chart of the whole experiment.

At 13:00 on the day of the experiment, participants arrived at the laboratory in a fed state (at least 2 h after their last meal). On arrival, their post-void nude body mass was recorded. An HR telemetry band (Polar Beat, Kempele, Finland) was fitted to the participant's chest. Temperature sensors were attached to four places on the skin surface (chest, upper arm, thigh, and leg) on the left side of the body to calculate the weighted mean skin temperature. Afterward, participants inserted a rectal thermistor 10 cm from the anal sphincter to measure T_c_. Then, at 13:55, after the participants were quietly seated on the ergometer, temperature sensors were connected to the data logger. Participants were then given equal volumes of either a placebo (trial C) or a GABA beverage (trial G). At 14:00, the experiment started, during which the temperature measurement software was used to continuously monitor the rectal temperature, skin temperature, and HR. It has been reported that plasma GABA concentrations decrease after oral administration of GABA, peak 20-40 min after ingestion, and remain significantly higher than baseline for at least an hour after ingestion (17). After a 20-min rest, at 14:20, participants performed continuous exercises at 60% of maximum output with a pedaling frequency of 60 rpm. At 14:40, participants were offered a sports drink to prevent dehydration (3 ml/kg body weight) while they remained seated on the ergometer. At 15:00, the exercise is completed, followed by a 20-min recovery period. The final body mass measurement was taken immediately after the exercise. The total time included 20 min rest plus 40 min exercise for the measurement plus 20 min resting time, giving a total of 80 min for the measurements. Each participant spent a total time of 140 min in the lab. The experimental protocol for this study is shown in Figure 1.

### Measurements

**Figure 2:**
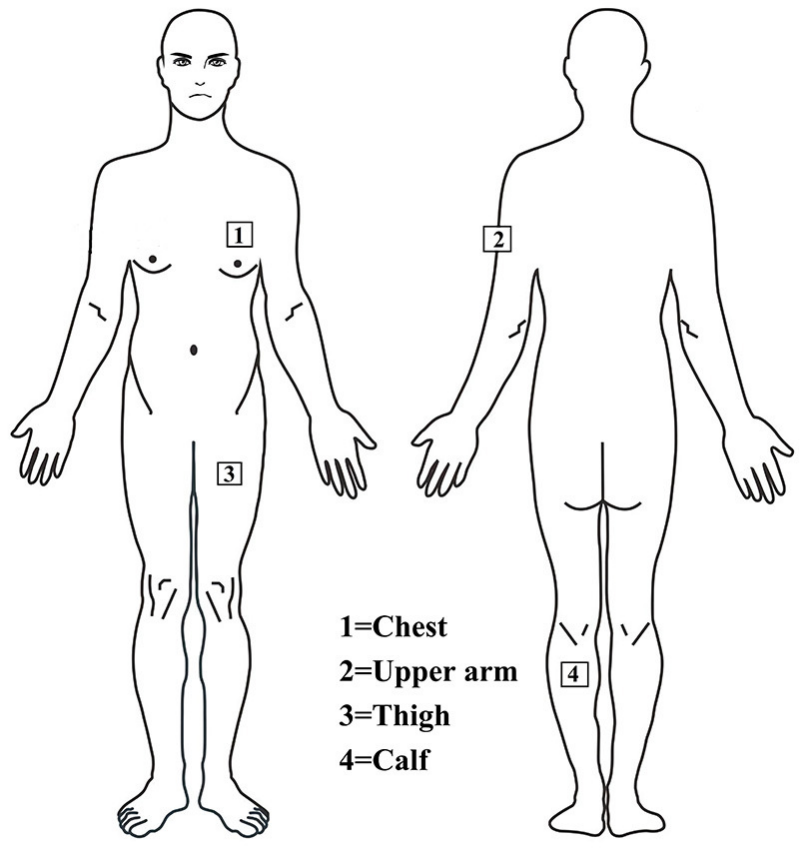
Thermistor probes are installed on the chest, upper arm, thigh and leg to measure skin temperature.

The main outcome measures of thermoregulation were core body temperature (T_c_), chest skin temperature (T_chest_), upper arm skin temperature (T_arm_), thigh skin temperature (T_thigh_), calf skin temperature (T_calf_), and HR. T_c_ (YSI401AC, YSI Precision^TM^, USA) and skin temperature (YSI409AC, YSI Precision^TM^, USA) probes were connected to a data logging device, and data were recorded every minute (Squirrel Meter Logger, Grant Instruments, Cambridge, UK). Skin thermistors were attached to four sites on the left side of the body using transparent Tegaderm patches surgical tape (3M, UK), shown in Figure 2. The same investigator applied probes to the skin to ensure consistent pressure was applied to each spot and each person. Pen markings provided reference points for each location. The mean skin temperature (T_sk_) was calculated using Ramanathan's proposed formula: T_sk_= 0.3 (T_chest_ + T_arm_) + 0.2 (T_thigh_ + T_calf_) (26). In addition, HR was measured in minute intervals throughout the experiment using polar telemetry (Polar Electro Oy, Kempele, Finland), and environmental conditions were recorded with a thermometer (JXBS-3001-TH-5-U, China). Estimated total sweating volume was derived by measuring the change in dry body mass (BM), measured immediately before and after the trial as (BM before) - (BM after) + 3 ml/kg (weight of sports drink). Dehydration was defined as a body mass loss of 2% or more (27).

### Statistical analysis

The data were analyzed using SPSS version 23.0 (Chicago, IL, USA), and the values were expressed as mean ± standard deviation. First, each outcome variable was examined for normality and sphericity. The one-sample Kolmogorov-Smirnov test for normal distribution was used, and log transformation was used for statistical analysis when the data were not normally distributed. When the assumption of sphericity was violated, the Greenhouse-Geisser correction was applied to reduce the probability of a type I error. We used a 2 [GABA-containing drink and placebo sport drink conditions) ×5 (rest (T_0_), exercise onset (T_20_), exercise (T_40_) 20 min, the exercise ends (T_60_), and recovery (T_80_)] analysis of variance (ANOVA) with repeated measures to assess the changes in mean HR, T_c_, and T_sk_ values. If a significant time × condition interaction was revealed, *post hoc* analysis was conducted using paired *t*-tests with the Bonferroni stepwise correction method to identify the pairwise differences. Body mass and sweat loss were analyzed using a paired *t*-test to determine differences in pre-exercise and post-exercise. Effect sizes were reported as Cohen for *t*-tests and partial eta squared (Pη^2^) for ANOVA. Statistical significance was established as *p* < 0.05.

## Results

### Effect of GABA on T_c_ and T_sk_

**Figure 3:**
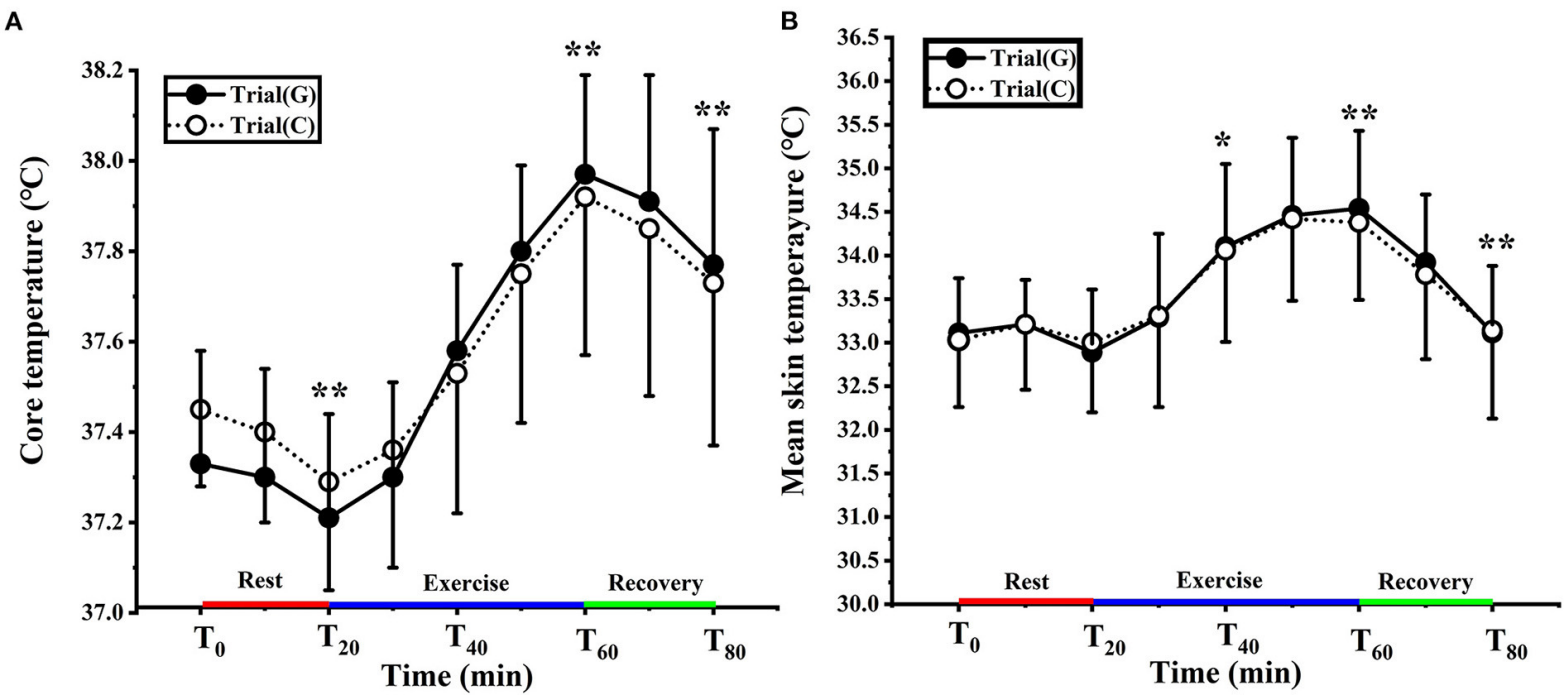
Change in core **(A)** and mean skin **(B)** temperature in trial G (black) and trial C (white). Values are means ± SD. Bars represent standard deviations.*P < 0.05, **P < 0.01, significant difference compared to T_0_.

As shown in Figure 3A, no significant interaction was noted [F (1.357, 24.434) = 1.181, *p* = 0.191, Pη^2^ = 0.091]. There was a significant main effect of time [F (1.357, 24.434) = 64.993, *p* < 0.01, Pη^2^ = 0.783]. Compared to T_0_, T_c_ decreased significantly at T_20_ and increased significantly at T_40_, T_60_ and T_80_ (*p* < 0.01). From 35–80 min, the T_c_ was higher in trial G (peaked at 37.96 ± 0.25°C) than in trial C (37.89 ± 0.37°C), but it failed to reach significant difference (*p* > 0.05).

There was no significant effect of GABA on T_sk_ [F (1, 18) = 0.015, *p* = 0.905, Pη^2^ = 0.001] and any condition × time interaction [F (1.941, 34.939) = 0.085, *p* = 0.914, Pη^2^ = 0.005], but there was a significant main effect of time [F (1.941, 34.939) = 35.431, *p* < 0.01, Pη^2^ = 0.663]. In trial G the T_sk_ of T_0_ was 33.12 ± 0.57°C, and for trial C, it was 33.24 ± 0.69°C (*p* > 0.05). T_sk_ was significantly higher than T_0_ at T_40_ (*p* < 0.05) T_60_ and T_80_ (*p* < 0.01). There was no significant difference between the two experiments (Figure 3B).

### Effect of GABA on skin temperature in different areas

**Figure 4:**
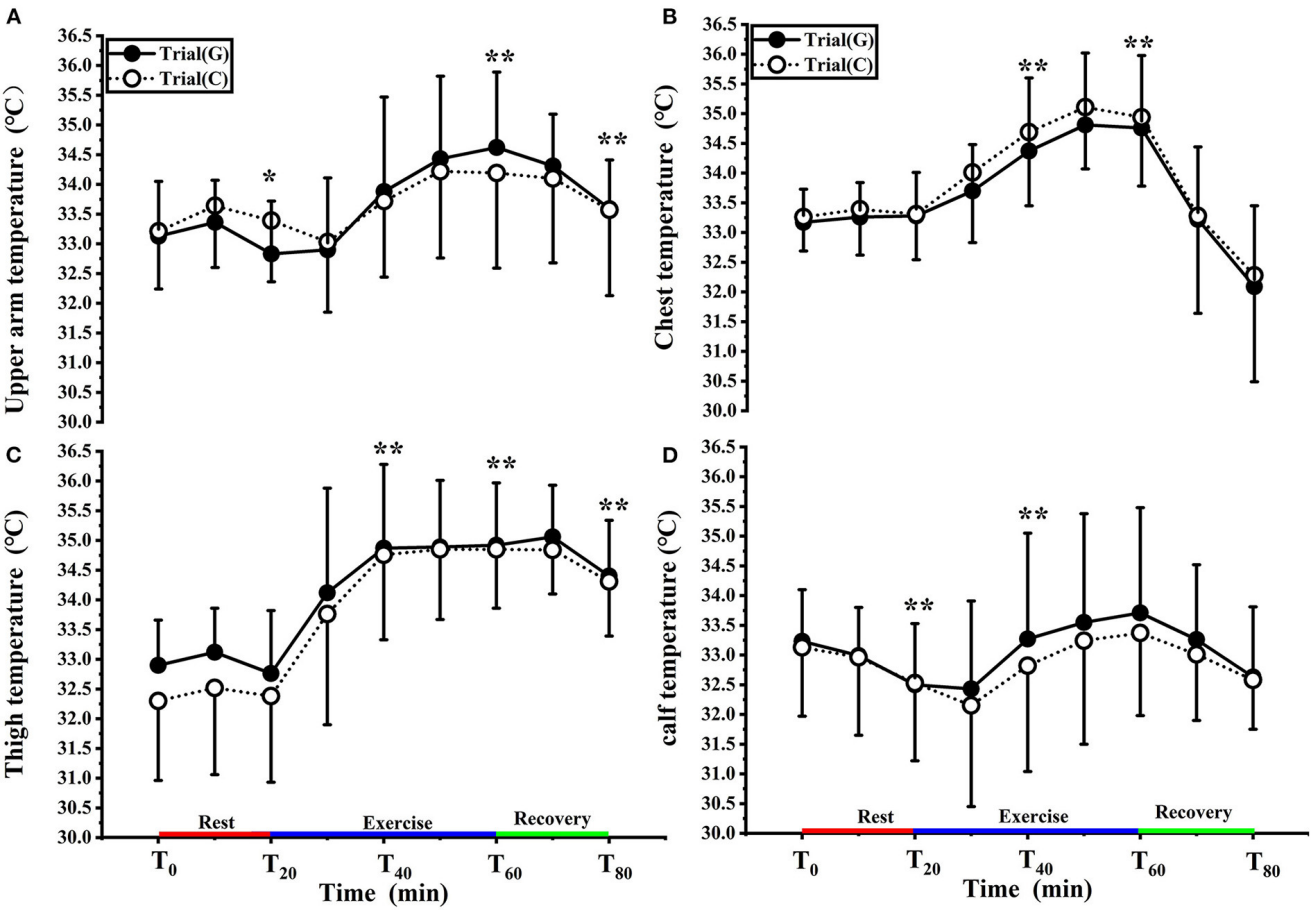
Change in upper arm **(A)**, chest **(B**), thigh **(C)**, calf **(D)** temperature in trial G (black) and trial C (white). Values are means ± SD. Bars represent standard deviations,*P < 0.05, **P < 0.01, significant difference compared to T_0_.

Figure 4A shows the changes in T_arm_ for both groups in the experiment. There was no significant effect of GABA on T_arm_ [F (1, 18) = 0.002, *p* = 0.996, Pη^2^ = 0.000] and no condition × time interaction [F (1.859, 33.468) = 0.572, *p* = 0.558, Pη^2^ = 0.031]. However, there was a significant main effect of time [F (1.859, 33.468) = 10.081, *p* < 0.01, Pη^2^ = 0.359]. At T_0_, T_arm_ was comparable between trial G (33.13 ± 0.92°C) and trial C (33.21 ± 0.97°C) (*p* > 0.05). Furthermore, the increase in T_arm_ over time was greater in trial G than trial C during exercise but the difference was not statistically significant (*p* > 0.05).

As shown in Figure 4B, there was no significant effect of GABA on T_chest_ [F (1, 17) = 0.039, *p* = 0.846, Pη^2^ = 0.002], and no condition × time interaction [F (1.741, 29.604) = 0.170, *p* = 0.815, Pη^2^ = 0.010]. However, there was a significant main effect of time F (1.741, 29.604) = 24.737, *p* < 0.01, Pη^2^ = 0.593). At T_0_, T_chest_ value of trial G and trial C were 33.17 ± 0.59°C, 33.26 ± 0.60°C respectively. During exercise, T_chest_ values increased and were significantly higher than T_0_ at T_40_ and T_60_ ( *p* < 0.01). T_chest_ values in trial G were lower than those in trial C (*p* > 0.05).

As shown in Figure 4C, there was no significant effect of GABA on T_thigh_ [F (1, 18) = 0.478, *p* = 0.498, Pη^2^ = 0.026], and no condition × time interaction [F (2.238, 40.284) = 0.897, *p* = 0.426, Pη^2^ = 0.047] was noted. However, there was, again, a significant main effect of time [F (2.238, 40.284) = 64.016, *p* < 0.01, Pη^2^ = 0.781]. Compared with T_0_, the T_thigh_ values of both groups increased in T_20_-T_40_, T_40_-T_60_ entered the platform stage during exercise (*p* < 0.01), and decreased after 10 min of recovery (*p* < 0.01); at each time point, there was no significant difference between the two trials.

As shown in Figure 4D, there was no significant effect of GABA on T_calf_ [F (1, 18) = 0.101, *p* = 0.754, Pη^2^ = 0.006] and no condition × time interaction [F (1.665, 29.965) = 0.230, *p* = 0.755, Pη^2^ = 0.013]. Again, a significant main effect of time [F (1.665, 29.965) = 4.352, *p* < 0.05, Pη^2^ = 0.195] was found. T_calf_ values decreased at T_20_ (*p* < 0.01); it rose after 10 minutes of exercise, was significantly higher than T_0_ at T_40_ (*p* < 0.01), and decreased during the recovery period. There was no significant difference between the two trials.

### Effect of GABA on HR

**Figure 5:**
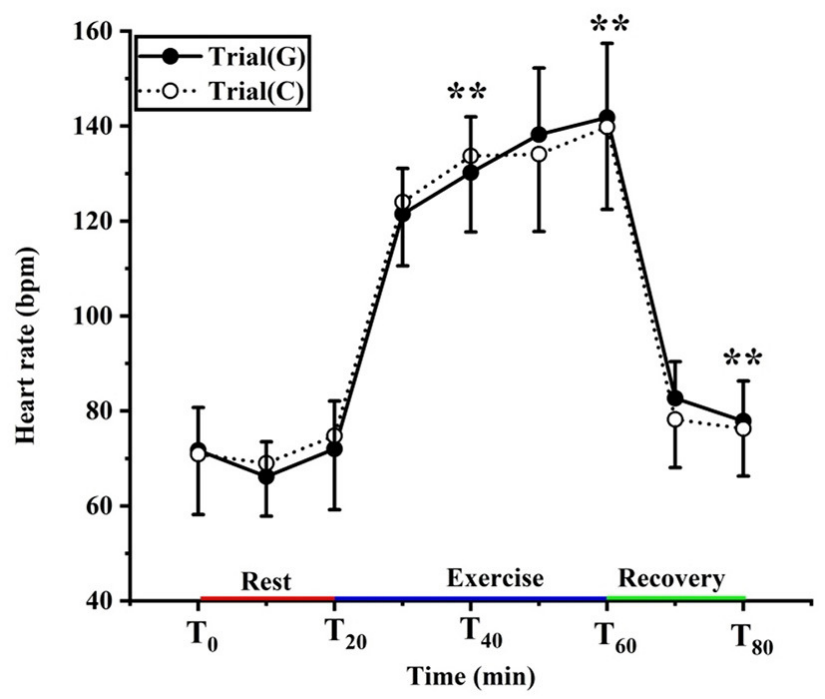
Changing in heart rate in trial G (black) and trial C (white). Values are means ± SD. Bars represent standard deviations, **P < 0.01, significant difference compared to T_0_.

There was no significant effect of GABA on HR (F (1, 18) = 0.006, *p* = 0.938, Pη^2^ = 0.000) and no condition × time interaction (F (1.419, 25.535) = 0.023, *p* = 0.589, Pη^2^ = 0.023). There was a significant main effect of time (F (1.419, 25.535) = 257.104, *p* < 0.01, Pη^2^ = 0.935). During exercise, HR increased over time (*p* < 0.01) and peaked at 141.80 ± 15.63 b/min with GABA drink and 139.80 ± 17.35 b/min with the placebo drink (*p* > 0.05) (Figure 5).

### The dehydration of the participants in the experiment

Compared to the pre-exercise weight, there was no significant difference in both groups. There was no difference between the two groups in sweating volume and dehydration rate (*p* > 0.05). The dehydration rate was <1%, indicating that no mild dehydration occurred in the participants of the study (Table 1).

**Table 1:** Changes in sweat volume and dehydration rate of the participants ($\bar{x}$ ± s, *n* = 10).

**Conditions**	**Pre-exercise weight (kg)**	**Post-exercise weight (kg)**	**Sweat volume (kg)**	**Dehydration rate (%)**
**Trial G**	**74.45 ± 5.26**	**74.46 ± 5.26**	**0.22 ± 0.11**	**0.29 ± 0.14**
**Trial C**	**74.05 ± 5.71**	**74.04 ± 5.76**	**0.23 ± 0.15**	**0.31 ± 0.21**

## Discussion

To the best of our knowledge, no study has previously explored the effects of oral administration of GABA on exercise thermoregulation responses of individuals in a cold environment. The main finding of this study was that oral administration of GABA did not influence the thermoregulation responses of individuals who exercise at cold temperatures. The thermoregulation parameters of T_c_, T_sk_, HR, and sweat loss did not differ between experimental and control groups.

### The effect of GABA on human thermoregulation during rest

At rest, the main challenge for human thermoregulation comes from cold ambient temperature conditions. The goal of body thermoregulation is to maintain core temperature by both feedback and feedforward mechanisms impinging on the integrative circuitry of the PO/AH (28). Maintaining a stable core temperature relies on balancing the heat generated and lost from conduction, convection, radiation, and evaporation (29). The cool sensation that elicits the cold defense response involves the activation of the cation channel conductance of the transient receptor potential 8 (TRPM8) in the skin's cold sensation thermoreceptors. The integrating center of the PO/AH receives signals from thermoreceptors in the skin that generate a typical physiological response to cold environments, including muscle shivering and cutaneous vasoconstriction-preventing heat loss and generating heat (29–31).

In our results, the values of T_c_ at T_0_ in trial C and trial G were 37.45 ± 0.17°C, and 37.33 ± 0.25°C, respectively. The changes in the T_sk_, T_chest_, and T_thigh_ were relatively small, while T_arm_ and T_calf_ both decreased consistently with time (Figures 3, 4). This finding concurs with Kruk et al. (32), who concluded that pre-exercise cold exposure resulted in a significant decrease in rectal temperature. With the increased temperature gradient between the body and the environment, the skin acts as a heat radiator for the body, and when the skin temperature is higher than the air temperature, heat is transferred from the body to the environment. As heat dissipation increases, the decrease in core body temperature causes the activation of mechanisms that preserve or generate heat. Skin temperature is controlled by blood flow to the skin surface, and when reacting to a cold environment the blood flow to the skin surface decreases so that less heat is transferred from the body core to the skin surface. Thus, the vasoconstrictive response to exposure to cold causes a decrease in heat dissipation and guarding of core temperature, resulting in a decrease in shell temperature. Repetitive rhythmic muscle contractions produce shivering to release heat, usually starting with contractile activity or redistribution of blood from the trunk and skeletal muscles, followed by blood flow to the distal part of the extremities (33). There were no significant differences in the values of T_c_ and T_sk_ between the groups in our studies. This may have been because oral administration of GABA was not yet effective within 20 min, or chemical stimulation did not affect the body temperature of GABAergic cells in the medial PO/AH (34, 35).

### The effect of GABA on human thermoregulation during exercise

The hypothalamus is the center of thermoregulation with the PO/AH integrating temperature information from all parts of the body and regulating body temperature by suppressing heat production in other parts (16). GABA is widely distributed in the assorted hypothalamic nuclei, especially in the PO/AH, and has a regulatory role in the body's temperature (36). There are two types of temperature-sensitive neurons in the PO/AH – heat-sensitive neurons and cold-sensitive neurons-controlling heat loss and heat generation, respectively (37). Animal studies show that GABA and GABA-A receptors in the PO/AH receive cold information from the skin and induce a thermogenic response (38).

Exercise increases heat output. However, a small portion of the heat generated becomes mechanical energy and is mostly dissipated as heat. The study results showed that the T_c_ of trial G and trial C continued to increase by 0.63 ± 0.31, 0.56 ± 0.35 respectively, until the end of the exercise. The T_c_ values in trial G tended to be higher than those of trial C from 35 to 60 min (Figure 3A). This suggests that both groups' gradual increase in core temperature was because of increased heat production by skeletal muscle and the liver. Heat thermoreceptors transmit signals to the PO/AH, which increases heat dissipation by stimulating vasodilation and sweat secretion in the skin. The skin is the body's main radiator and circulating blood transfers heat from the core to the skin surface to dissipate heat.

In our study, both groups' T_sk_, T_arm_, and T_calf_ increased during exercise (Figures 3B, 4A,D). In trial G, the value of T_arm_ and T_calf_ was higher than trial C. This may have been because the skin vasodilates and more blood flows to the skin surfaces, distributing blood to the shell in exercise. It was reported that forearm blood flow increases rapidly with core temperature in humans (39). Another study showed that people who exercise regularly did not have reduced leg blood flow during submaximal performance cycling (40). In a study by Galloway (41), rectal temperature reached 39.2°C and mean skin temperature decreased from 25°C to 22.5°C when individuals cycled at 70% VO_2max_ at an ambient temperature of 11°C. Participants performing sustained exercise at moderate intensities may be less likely to have decreased rectal temperatures in an environment of 3–11°C (42). Another study demonstrated that with temperatures between −10°C and 20°C, there was no difference in rectal temperature when individuals exercised at 64% VO_2max_, suggesting an elevated core temperature separate from ambient temperature (43). These studies indicate that exercise intensity is sufficient to prevent a decrease in core temperatures and that cold-sensitive receptors in the PO/AH are not activated, resulting in the inability of GABA to function.

Hyperthermia, with a core body temperature above 38°C, is thought to be an important factor contributing to central fatigue and limiting endurance exercise capacity (44). However, there were no significant differences in core and mean skin temperatures between the groups during exercise. GABA consumption did not significantly increase core body temperature, and thus the risk and negative effects of hyperthermia during exercise were reduced. It is also possible that oral GABA raises core temperature under cold conditions and then lowers core temperature because of increased heat production from exercise. The two effects of GABA would then be largely neutralized, maintaining core temperature in the normal range rather than further raising it. These findings suggest that GABA is a safe food supplement ingredient even when used in cold conditions during exercise.

Some animal studies suggest that GABA may not be able to cross the blood-brain barrier (BBB) (45, 46), which may explain the lack of modulatory effect of GABA administration in the present experiment. Nevertheless, additional evidence suggests that small concentrations of GABA can reach the BBB (47, 48). This should be clarified in future studies using nuclear magnetic resonance spectroscopy (MRS) in humans to study the effect of GABA administration on GABA concentration. While we recognize the limitations of our work, animal experiments have used an ambient temperature nearly 5°C colder than the present experiment. The ambient temperature in our study may have been insufficiently cold to stimulate cold-sensitive neurons in the preoptic-thalamic anterior, yielding inconsistent results. A possible explanation for GABA's limited effect on the core body and mean skin temperature could be the relatively moderate ambient conditions in which the exercise tests were performed. Exercise at an ambient temperature of 10°C suggests that the exercise stimulus was sufficient to balance heat generation and heat dissipation for both groups. The pro-cooling mechanism of increasing heat storage was sufficient for the participants to overcome core temperature increases during exercise, like increased skin heat dissipation during exercise in the cold.

### The effect of GABA on human thermoregulation during recovery

Both groups showed a linear decrease in core body temperature and mean skin temperature after exercise during the recovery period, with values higher than baseline values, implying that participants did not recover sufficiently. However, core body temperature and mean skin temperature were higher in trial G than in trial C and some parts of the skin temperature decreased. However, T_c_ and T_sk_ did not differ between the groups. It may be that at the end of the exercise, the core body temperature deviated from the value of the turning point. The thermoregulatory system then started to work, and the heat-sensitive neurons received a signal from the PO/AH leading to stimulation of the heat signal and more impulses being sent. This promoted sweat secretion through neurotransmission, increasing blood flow to the skin and promoting heat dissipation, resulting in lower body temperature. The preoptic areas of the hypothalamus react to heat and the medial dorsal area to cold, with a degree of antagonism between these two brain regions (49). When heat production increases, activation of neurons in the preoptic area inhibits the response to cold during exercise. Therefore, there was no increase in core body temperature for exercising participants administered GABA orally.

### The effect of GABA on human HR

Heart rate is also an essential indicator in the process of thermoregulation. During exercise, the core temperature rises due to the movement of skeletal muscles, and the body needs to dissipate heat through the skin, so the blood flow to the skin is enhanced, leading to an increase in heart rate. It has been reported that within a certain range, for every 2°C increase in body temperature, cardiac output increases by ~10%, and peripheral resistance decreases by 20%. During the transition from rest to exercise, the rapid increase in heart rate (HR) predominantly mediated by cardiac vagal withdrawal, supported by increased cardiac sympathetic activity after a few seconds (50, 51). This experiment showed that after exposure to 11°C for 20 min, both groups of the heart rate compared to 0 min increased. During exercise, the heart rate significantly increased in trials G and C, peaking at 141.80 ± 15.63 b/min, 139.80 ± 17.35 b/min, respectively. When the athletes is exercising in the cold, blood is redistributed from the central part of the body to the peripheral tissues to increase heat dissipation from the skin, cardiovascular demands may dramatically increase due to the dual demand for blood supply by the working muscles and the skin, which in turn increases the heart rate. After exercise, the heart rate maintains stability after a rapid descent to pre-exercise level. However, there were no differences among groups in heart rate. Upon entering the recovery period, increased venous reflux caused baroreceptor reflex.

HR decreased rapidly in our study unlike previous research that showed GABA activation in conscious rats induces press or response and tachycardia (52, 53). However, the extent to which these findings can be extrapolated to humans remains unclear. Previous studies have indicated that central GABAergic mechanisms are involved in the HR responses at the onset of exercise. Teixeira (54) studied participants randomly performing three bouts of 5s passive and active cycling under placebo and oral administration of diazepam (10 mg). The GABAergic mechanisms contributed significantly to the muscle mechanome reflex-mediated HR responses at the onset of exercise. Possible explanations for this may be that diazepam crosses the BBB (55). Contrary to Teixeira's (54) findings, we did not find that GABA affects HR. The results of studies on whether GABA can cross the BBB are inconsistent, and the findings in our study are likely because GABA cannot cross the BBB.

### Dehydration of participants in the experiment

Sweat production and the subsequent evaporation are the principal modes of heat loss in humans during exercise. Exercise elevates core and skin temperatures, contributing to the increased sweat rate. When the body core temperature rises above the critical level of 37°C (98.6 F), sweating increases the rate of heat loss. This experiment was conducted before and during exercise with 3 ml/kg body weight each time. By measuring body weight before and after exercise, we concluded that the rate of sweat loss between the experimental and control groups did not reach the level of dehydration, and there was no significant difference between the two groups, indicating that the exercise capacity of the participants was not affected by dehydration in the two exercise groups.

## Conclusion

There are several limitations to this study. First, although we estimated an appropriate sample size using the G-power software, the number of participants only met the minimum sample size requirement. Second, Only 1,000 mg of GABA was validated for its effect on thermoregulation in cold environments. In future studies a larger sample size should be used to study the effect of different doses of GABA intake for prolonged exercise on thermoregulation and reveal the dose-effect relationship. Finally, it has been reported that serum GABA concentrations peak at 20–40 min and can be maintained at higher than pre-dose levels for at least 1 h. The present experiment involved a rest period of 20 min, but it is possible that plasma GABA concentrations vary in each individual. In the present study, we did not measure plasma GABA concentrations. Therefore, future research should measure plasma GABA levels and clarify the mechanisms involved in conjunction with animal experiments.

Oral administration of GABA was not shown to be effective in increasing core body temperature of individuals when cycling for 40 min in a cold environment, but it is a safe supplemental ingredient for exercise and other healthy diets. Core temperature can be increased by exercise for a short period of time in cold environments. However, it is important to wipe sweat and keep warm after exercise to avoid upper respiratory tract infections. Whether different doses of GABA have any potential effect on thermoregulation during exercise of longer duration in cold environments requires further investigation.

## Data availability statement

The datasets presented in this study can be found in online repositories. The names of the repository/repositories and accession number(s) can be found in the article/Supplementary material.

## Ethics statement

The studies involving human participants were reviewed and approved by Guangxi Normal University. The patients/participants provided their written informed consent to participate in this study.

## Author contributions

HW conceived and supervised the study. YH and HW designed the experiments and carried out the experiments. LC analyzed the data and wrote the manuscript. All authors have read and agreed to the published version of the manuscript.

## Funding

This study was supported by the Innovation Project of Guangxi Graduate Education (No.YCBZ2022069).

## Conflict of interest

The authors declare that the research was conducted in the absence of any commercial or financial relationships that could be construed as a potential conflict of interest.

## Publisher's note

All claims expressed in this article are solely those of the authors and do not necessarily represent those of their affiliated organizations, or those of the publisher, the editors and the reviewers. Any product that may be evaluated in this article, or claim that may be made by its manufacturer, is not guaranteed or endorsed by the publisher.
